# Mitochondrial Dysfunction in Sickle Cell Trait Carriers With Exertional Collapse

**DOI:** 10.1155/crig/4478581

**Published:** 2025-10-07

**Authors:** Kristen A. Cofer, Liam Friel, Mingqiang Ren, Carolyn Dartt, Francesca Cariello, Kyung Kwon, Patricia A. Deuster, Nyamkhishig Sambuughin, Tianzheng Yu, Francis G. O'Connor

**Affiliations:** ^1^Consortium for Health and Military Performance, Department of Military and Emergency Medicine, F. Edward Hébert School of Medicine, Uniformed Services University, Bethesda, Maryland, USA; ^2^Henry M. Jackson Foundation for the Advancement of Military Medicine, Inc., Bethesda, Maryland, USA

**Keywords:** exertional injury, mitochondrial depletion, pathogenic variant, rhabdomyolysis, sickle cell trait

## Abstract

Sickle cell trait (SCT) increases the risk of sudden death and exertional rhabdomyolysis (ER) in athletes and Service Members (SMs) during intense exercise. Exertional injuries in SCT carriers can result in exercise collapse associated with SCT (ECAST), an under-recognized condition characterized by variable clinical presentations ranging from ischemic muscle pain to fulminant collapse. This study presents clinical and genetic findings of two independent Black SMs with history of ECAST triggered by strenuous exercise. ECAST cases carried pathogenic heterozygous mutations *POLG*: p.Gly848Ser and *RRM2B:* p.Met282Ile associated with mitochondrial DNA depletion syndromes. Mononuclear cell mitochondria extracted from ECAST cases showed impaired mitochondrial profiles and resilience, demonstrating the potential contribution of mitochondrial dysfunction to exertional collapse in SCT carriers.

## 1. Introduction

Approximately 300 million people worldwide and nearly 7.8% of African Americans in the United States have sickle cell trait (SCT), a carrier state of mutant hemoglobin (HbAS) that in HbSS form causes sickle cell disease [1–3]. While generally considered benign, SCT has been associated with exertional injuries during intense physical exertion, especially in hot climates or high-elevation locations, or when dehydrated [1]. Exercise collapse associated with sickle cell trait (ECAST), a potentially life-threatening condition in SCT carriers, is becoming more widely recognized by the medical community. ECAST can present during or following a significant exertional effort, with unusual muscle weakness and pain, normal to modest elevation of core temperature, and an initial conscious collapse without signs of central nervous system dysfunction [4]. Although the absolute number of athletes and Service Members (SMs) who die from ECAST is extremely low, the association between sudden unexplained deaths and SCT in athletes and SMs has been increasingly recognized [4, 5]. In a study investigating the cause of sudden death cases in National Collegiate Athletic Association student athletes (2004–2008), researchers reviewed 2 million athlete years and found that African American football players with SCT had a significantly higher risk of mortality from exertion than those without SCT [5]. Before implementing safety precautions, this statistic was similar among military recruits [4, 5].

While the clinical description of ECAST is well defined, its etiology remains unclear. Existing research has focused attention on blood disorders, including alpha thalassemia and red cell pyruvate kinase deficiency [6]. This report presents two ECAST cases with pathogenic variants in nuclear genes linked to mitochondrial DNA depletion syndromes (MDS), suggesting impaired energy expenditure may contribute to exertional complications in SCT carriers.

## 2. Methods

This study protocol was approved by the Uniformed Services University Institutional Review Board. Four SCT-positive participants, ages 26–30 and of Black racial background, were recruited and categorized as cases (history of exertional collapse; *n* = 2) or controls (no history of exertional collapse; *n* = 2). All participants provided informed consent and completed a questionnaire detailing demographics, physical characteristics, behaviors, and medical/family histories. Cases underwent additional review of electronic medical records and a physician phone interview to gather specific exercise collapse details. Blood samples were collected from all participants for whole exome sequencing (WES) and mitochondrial profiling.

### 2.1. Specific Tests

#### 2.1.1. WES

WES was performed as previously described [7]. Briefly, nonsynonymous, splice, stop gain, and stop loss variants were annotated and prioritized with minor allele frequency filtering criteria of ≤ 0.01. Variants classified as pathogenic (P), likely pathogenic (LP), variant of uncertain significance (VUS), and variants with no annotation (NA) in ClinVar (https://www.ncbi.nlm.nih.gov/clinvar) were analyzed.

#### 2.1.2. Measurement of Mitochondrial Functions in Human Peripheral Blood Mononuclear Cells (PBMCs)

Human PBMCs were isolated from whole blood using a standard density gradient centrifugation technique, resuspended in RPMI-1640 medium. Mitochondrial oxygen consumption rate (OCR) was determined by measuring the O_2_ concentration with a Clark-type O_2_ electrode [8]. Briefly, after a basal OCR was recorded, mitochondrial proton leak was determined by adding 2-μM oligomycin (ATP synthase inhibitor). Maximal uncoupled respiration was determined by adding 2-μM carbonyl cyanide p-trifluorome-thoxyphenylhydrazone (FCCP; uncoupler), and nonmitochondrial respiration was determined by adding 1-μM rotenone (complex I inhibitor) and antimycin A (complex III inhibitor). At the end of each experiment, 5-μM rotenone was added to the PBMCs to confirm that decreases in the O_2_ concentration originated from mitochondrial respiration. A mitochondrial resilience index was calculated by dividing ATP-linked OCR and reserve capacity by proton leak and nonmitochondrial, as follows [9]:

(1) $$\begin{array}{c} \text{Mitochondrial resilience index}=\frac{\text{ATP}-\text{linked OCR}\times \text{reserve capacity}}{\text{proton leak}\times \text{nonmitochondrial OCR}}\times 100. \end{array}$$

## 3. Results

Demographic data and ECAST event presentation of participants are presented in Table 1.

### 3.1. Case A

**Figure 1:**
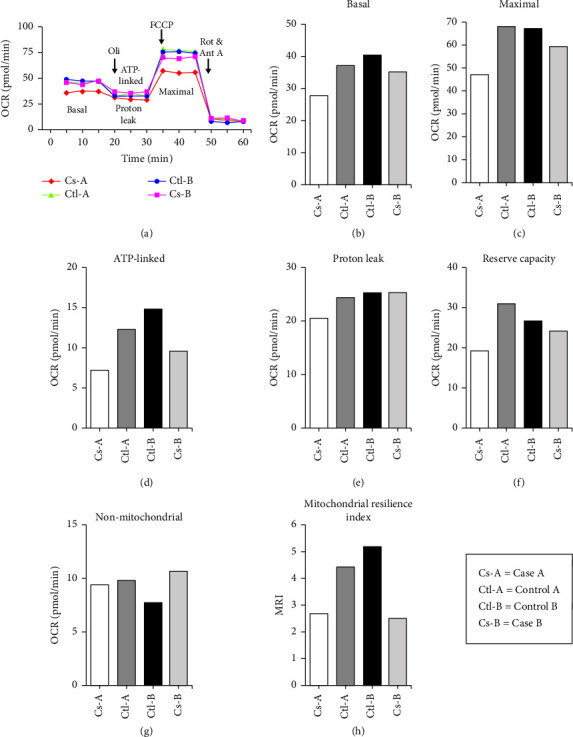
Summary of blood cell mitochondrial profile. (a) Representative profiles of mitochondrial biogenesis in peripheral blood mononuclear cells using inhibitors, oligomycin (Oli), FCCP, rotenone (Rot), and antimycin (Ant A). (b) Basal measured before the injection of mitochondrial inhibitors. (c) Maximal capacity of the mitochondrial electron transport system with the presence of its uncoupler (FCCP). (d) ATP linked determined by the difference between basal and respiration following Oli exposure. (e) Proton leak determined by the remaining rate of mitochondrial respiration with the presence of Oli, which is an inhibitor of ATP synthase. (f) Reserve capacity determined by the difference between the basal and maximal. (g) Nonmitochondrial calculated by the remaining respiration following the addition of the complex inhibitors Rot and Ant A. (h) Calculated by dividing ATP linked and reserve capacity by proton leak and nonmitochondrial.

A SM collapsed near the completion of a three-mile run. Based on presenting symptoms and peak creatine kinase, he was diagnosed with rhabdomyolysis, compartment syndrome of the left buttock, left thigh, and bilateral lower leg anterolateral compartments, and acute renal failure requiring hemodialysis. Surgical intervention included fasciotomies of the left and right lower legs, left thigh, and left buttock. WES identified heterozygous pathogenic variant p.Gly848Ser (NM_002693.3: c.G2542A, rs113994098) in the *POLG* gene, associated with MDS [10]. Mitochondrial profiling (Figures 1(a), 1(b), 1(c), 1(d), 1(e), 1(f), and 1(g)) showed the following OCRs in picomoles per minute (pmol/min): basal OCR = 27.67; maximal OCR = 46.67; ATP-linked OCR = 7.33; proton leak = 20.33, reserve capacity = 19; and nonmitochondrial OCR = 9.3. The mitochondrial resilience index was determined to be 2.65 (Figure 1(h)).

### 3.2. Case B

A SM collapsed during an indoor physical fitness test. He had a history of childhood reactive airway disease but reported no recent symptoms. WES revealed heterozygous pathogenic variant p.Met282Ile (NM_015713.5:c.846G > C, rs182614164) in the *RRM2B* gene, associated with MDS [10]. Mitochondrial profiling (Figures 1(a), 1(b), 1(c), 1(d), 1(e), 1(f), and 1(g)) showed the following OCRs (pmol/min): basal OCR = 35, maximal OCR = 59.33, ATP-linked OCR = 9.67, proton leak = 25.33, reserve capacity = 24.33, and nonmitochondrial OCR = 10.67. The mitochondrial resilience index was determined to be 2.49 (Figure 1(h)).

### 3.3. Control A

This civilian had no history of ECAST. Genetic testing for mitochondrial disorders, including MDS, was negative. Mitochondrial profiling (Figures 1(a), 1(b), 1(c), 1(d), 1(e), 1(f), and 1(g)) showed the following OCRs (pmol/min): basal OCR = 36.67; maximal OCR = 67.67; ATP-linked OCR = 12.33; proton leak = 24.33, reserve capacity = 31; and nonmitochondrial OCR = 9.67. The mitochondrial resilience index was determined to be 4.43 (Figure 1(h)).

### 3.4. Control B

This SM had no history of ECAST. Genetic testing for mitochondrial disorders, including MDS, was negative. Mitochondrial profiling (Figures 1(a), 1(b), 1(c), 1(d), 1(e), 1(f), and 1(g)) showed the following OCRs (pmol/min): basal OCR = 40.33; maximal OCR = 67.33; ATP-linked OCR = 15; proton leak = 25.33; reserve capacity = 27; and nonmitochondrial OCR = 7.67. The mitochondrial resilience index was determined to be 5.17 (Figure 1(h)).

## 4. Discussion

SCT is largely considered a benign condition. However, during intense physical exertion, in particular when accompanied by environmental heat and/or altitude, SCT carriers may be at risk for ECAST [1]. While the underlying mechanisms are not well known, current research suggests exertional sickling and/or genetic factors are contributing factors [9, 11, 12]. In this case report, we describe two SCT carriers presenting with exertional collapse events, both of whom demonstrated associated pathogenic variants linked to MDS, suggesting the potential contribution of impaired energy expenditure in ECAST.

Remarkably, two independent ECAST cases carried pathogenic mutations in the *POLG* and *RRM2B* genes. The *POLG* and *RRM2B* genes are associated with MDS [10]. MDS are genetically and clinically heterozygous autosomal recessive disorders defined by a significant reduction in mtDNA content, resulting in impaired energy production in the affected tissues and organs [10]. The *POLG* gene is responsible for encoding mtDNA polymerase, the enzyme that replicates the mitochondrial genome [10]. The *RRM2B* gene encodes ribonucleotide reductase M2 B subunit, the enzyme essential for DNA synthesis and to maintain a balanced mitochondrial nucleotide pool [10]. Mutations in both genes are also presented with a myopathic phenotype and reduced mitochondrial content in skeletal muscles [13]. While carriers of autosomal recessive mutations are typically asymptomatic, identification of two ECAST cases with similar phenotypes and carrying pathogenic mutations associated with MDS is significant. In alignment with this, the mitochondrial profiling showed that both cases exhibited impaired mitochondrial functions. Healthy mitochondria should exhibit high reserve capacity, high ATP-linked OCR, and low proton leak [9]. Conversely, our cases demonstrated low reserve capacities and low ATP-linked OCRs compared with the controls. Reduced reserve capacity may result from oxidative stress, and reduced ATP-linked OCR may indicate low ATP demand, substrate unavailability, and/or damaged oxidative phosphorylation [9]. To further differentiate between healthy and unhealthy mitochondria, the mitochondrial resilience index was calculated. Healthy mitochondria should exhibit a high mitochondrial resilience index, but both cases had lower mitochondrial resilience indices than the controls, further supporting the presence of mitochondrial dysfunctions [9].

Of note, the presence of HbS and thalassemia could represent potential sources of bias when comparing SCT carriers with and without exertional injuries. We did not measure HbS or normal hemoglobin subunit levels in this study, as our focus was on assessing mitochondrial function in PBMCs. Recent studies have shown that hemoglobin subunits can be expressed in PBMCs and may function as redox modulators [14]. Future studies are warranted to investigate the role of HbS in PBMCs and whether it significantly influences mitochondrial function. Regarding thalassemia, we performed WES on all four subjects, as described in the Methods section, and found no missense or synonymous variants in the exonic regions of the HBA1 and HBA2. All four participants carried the heterozygous HbS variant (rs344), but none carried additional exonic variants in the HBB gene.

In conclusion, we present two unrelated individuals with SCT who experienced ECAST events during exercise. Genetic analysis revealed pathogenic *POLG:*p.Gly848Ser and *RRM2B:*p.Met282Ile mutations, both associated with MDS. Mitochondrial profiling demonstrated poor mitochondrial efficiency in both cases, supporting genetic findings. While the pathophysiology of ECAST remains poorly understood, our findings suggest that genetic and mitochondrial bioenergetic analyses in individuals diagnosed with ECAST may provide valuable insight. Future research with a larger cohort should explore the role of mitochondrial dysfunctions and other genetic factors contributing to ECAST.

## Figures and Tables

**Table 1:** Demographics and clinical characteristics of participants.

	Case A	Case B	Control A	Control B
Demographics				
Gender	Male	Male	Male	Female
Age	26	30	29	28
ECAST event history				
Activity engaged in prior to event	Outdoor three-mile run	Indoor physical fitness test	NA	NA
Presentation	Alert and oriented; dyspnea; severe muscle cramping and pain in the legs and back; dark urine	Alert and oriented; dyspnea; muscle pain and weakness; diaphoresis, cool and clammy skin	NA	NA
Initial core temperature (°F)	103	Not obtained	NA	NA
Peak CK (U/L)	> 300,000	3103	NA	NA
Days hospitalized	24	0	NA	NA
Physical activity in past 12 months (days per week)	7	3–4	3–4	5–6
Prior history of ERE?	No	No	No	No
Family history of adverse reactions to anesthetics or neuromuscular disorders?	No	No	No	No

Abbreviations: CK = creatine kinase, ERE = exertion-related event, NA = not applicable.

## Data Availability

All relevant data are included within the manuscript. Further inquiries can be directed to the corresponding author.
